# Valva Culpada: Um Novo Conceito para Redução do Risco da Intervenção no Portador de Doença Valvar Grave

**DOI:** 10.36660/abc.20240492

**Published:** 2025-04-17

**Authors:** Roberto Cintra de Azevedo Aragão, José Augusto Soares Barreto, Roney Orismar Sampaio

**Affiliations:** 1 Universidade Federal de Sergipe São Cristóvão SE Brasil Universidade Federal de Sergipe, São Cristóvão, SE – Brasil; 2 Fundação São Lucas Centro de Ensino e Pesquisa Aracaju SE Brasil Fundação São Lucas, Centro de Ensino e Pesquisa, Aracaju, SE – Brasil; 3 Universidade Federal de Sergipe Programa de Pós-Graduação em Ciências da Saúde Aracaju SE Brasil Universidade Federal de Sergipe - Programa de Pós-Graduação em Ciências da Saúde, Aracaju, SE – Brasil; 4 Instituto do Coração do Hospital das Clínicas Faculdade de Medicina Universidade de São Paulo São Paulo SP Brasil Instituto do Coração do Hospital das Clínicas da Faculdade de Medicina da Universidade de São Paulo, São Paulo, SP – Brasil

**Keywords:** Doenças das Valvas Cardíacas, Prognóstico, Cardiologia

Na prática clínica, não é incomum nos depararmos com duas condições clínicas relativamente graves que ocorrem concomitantemente e podem justificar os sintomas do paciente. Por exemplo, as associações entre estenose aórtica e coronariopatia, ou entre estenose aórtica e insuficiência mitral primária ou secundária, ambas de grau moderado a importante, são corriqueiras no mundo real. Diante de pacientes com baixo risco cirúrgico, é consenso o tratamento combinado no momento da intervenção cirúrgica, a despeito das consequências desses procedimentos combinados, a exemplo do aumento da morbimortalidade cardiovascular. A título de exemplo, a troca da valva aórtica (VAo), combinada com a cirurgia de revascularização miocárdica, aumenta em quase duas vezes o risco de mortalidade, assim como a troca da VAo, combinada com o tratamento cirúrgico da valva mitral (VMi), aumenta em mais de três vezes esse risco.^1^ Entretanto, em pacientes que apresentam risco perioperatório elevado, especialmente em situações nas quais um procedimento menos invasivo isolado pode ser empregado, a tomada de decisão entre indicar um tratamento mais amplo, mas com maior morbimortalidade, comparado a um tratamento isolado, porém de menor morbimortalidade, não é trivial.

Recentemente, em discussões de Heart Team (HT), com o advento do tratamento da VAo pelo implante percutâneo da válvula aórtica (TAVI) ou mesmo do tratamento “borda a borda” e “valve in valve” da VMi, ganhou força a ideia de que o tratamento isolado da lesão mais grave, provável responsável principal pelos sintomas do paciente, tem potencial de otimizar o desfecho do paciente, reduzindo a morbimortalidade esperada e melhorando substancialmente a sua qualidade de vida.

Na síndrome coronariana aguda (SCA), é prática comum tratar prioritariamente a artéria responsável pelo evento agudo. Em um segundo momento, que pode ocorrer ainda na fase de internação hospitalar ou após a estabilização do quadro clínico, indica-se, ou não, a revascularização adicional de outras lesões coronarianas obstrutivas. Extrapolando-se para as doenças valvares, surge o conceito de “*valva culpada*” e o tratamento hierarquicamente prioritário desta. Em discussões de *Heart Team*, não é raro enfrentar situações desse tipo; sendo assim, o estabelecimento do conceito de “valva culpada”, tal como ocorre na SCA, pode trazer benefícios tangíveis para tais pacientes.

A doença valvar múltipla é uma condição altamente prevalente cuja incidência tem aumentado com o envelhecimento populacional. A condição é adquirida na maioria das vezes e está associada a uma piora no prognóstico.^2^ Além disso, em países onde a doença valvar reumática é prevalente, a incidência da doença multivalvar grave simultânea é ainda maior.^3^

Os métodos utilizados para a quantificação de estenose e regurgitação valvar foram validados para valvopatias isoladas, o que dificulta a análise de doença múltipla e impõe um desafio diagnóstico e terapêutico.^4^ As interações hemodinâmicas dependem de combinações específicas das lesões valvares, gravidade e tempo do início de cada lesão individual, condições de sobrecarga e desempenho ventricular sistólico. As principais condições que podem impactar no diagnóstico da doença valvar múltipla são: presença frequente da estenose de baixo fluxo e baixo gradiente; equação de continuidade inaplicável quando os fluxos transvalvares são desiguais; e lesões valvares graves, podendo induzir ou aumentar as regurgitações mitral e tricúspide secundárias a montante. Além disso, métodos derivados do tempo de meia pressão podem ser inválidos na presença de complacência/relaxamento alterado do ventrículo esquerdo.^2^

Como exemplo, a regurgitação mitral funcional está presente em mais de 60% dos pacientes com estenose aórtica. Ela reduz a pós-carga e o volume sistólico, podendo favorecer uma condição de menor fluxo e baixo gradiente, com risco de subestimar a gravidade da estenose aórtica. Simultaneamente, a presença de regurgitação mitral reduz a pós-carga total e aumenta a fração de ejeção, podendo ocultar a disfunção miocárdica subclínica. Nesses casos, a área da VAo aparenta ser o método mais confiável de avaliação.^4^

Diante desse quadro, a doença valvar múltipla pode ser de difícil interpretação, necessitando de multimodalidade de imagem e do *Heart Team* para melhor estratégia diagnóstica e manejo terapêutico, sobretudo com a progressiva evolução dos procedimentos por cateter.^2,4^

A cirurgia valvar combinada apresenta alta taxa de mortalidade hospitalar, sobretudo nos pacientes com disfunção ventricular esquerda, insuficiência renal e idade mais avançada, população em que não raro se observa a presença da doença valvar múltipla.^5-7^ A preferência do paciente também deve ser uma variável na escolha do caminho a ser seguido. Uma tomada de decisão que pondere, de forma mais holística, todos esses fatores tem o potencial real de proporcionar melhores resultados no curto, no médio e no longo prazos.

Na avaliação hemodinâmica pós-TAVI de pacientes com estenose aórtica grave e estenose mitral concomitante (área valvar mitral (AVM) ≤ 2,0 cm^2^), foi observado que metade dos pacientes melhoraram a AVM para além de 2,0 cm,^2^ permitindo inferir que esta havia sido superestimada (pseudoestenose mitral). Alguns preditores que sugerem a presença de estenose mitral verdadeira são: AVM ≤ 1,5 cm^2^, distensão do anel mitral ≤ 1 mm ou extensão da calcificação para os folhetos mitrais anterior e posterior.^8^ Diante disso, uma identificação mais adequada dos pacientes que podem se beneficiar do tratamento combinado pode reduzir a morbimortalidade e evitar tratamentos múltiplos desnecessários.

Por outro lado, não corrigir uma lesão valvar moderada/importante associada, situação cada vez mais comum em pacientes idosos com estenose aórtica, os quais são submetidos ao TAVI, requer uma integração de fatores e individualização. Entendimento da história natural, etiologia das valvas acometidas, expectativa de vida, comorbidades, risco cirúrgico, possibilidade de reparo e viabilidade de abordagem percutânea são informações indispensáveis para a tomada de decisão. As interações hemodinâmicas podem alterar a expressão clínica de cada lesão singular, logo, os médicos devem estar cientes dessas interações que podem impactar no diagnóstico. Todos esses fatores devem ser considerados na análise do Heart Team valvar.

Elderia et al. evidenciaram que uma abordagem intervencionista estagiada apresenta vantagem de sobrevivência no curto prazo em comparação com a cirurgia combinada para tratamento da doença arterial coronária e estenose aórtica.^9^ Um estudo recente mostrou que a intervenção coronária percutânea pós-TAVI não expõe os pacientes a maiores riscos periprocedimento e fornece uma tendência de resultados clínicos favoráveis em médio e longo prazos,^10^ mesmo se houver revascularização miocárdica incompleta.^11^ No entanto, apesar de a taxa de sucesso do acesso coronário pós-TAVI ser alta, a intervenção coronária percutânea (ICP) antes da TAVI deve ser considerada em pacientes com doença arterial coronária (DAC) grave proximal, particularmente na síndrome coronária aguda, sintomas de angina ou lesões suboclusivas, especialmente se a prótese selecionada for supra-anular. A previsão da viabilidade do acesso coronário futuro com base na avaliação de imagem tomográfica pré-TAVI é essencial para orientar a escolha do tipo de prótese e da altura do implante e realizar o alinhamento comissural, sobretudo em pacientes jovens e de menor risco.^12^

As evidências limitadas sobre o tratamento médico, cirúrgico e intervencionista no cenário da doença valvar múltipla são enfatizadas pelo nível C de evidência na maioria dos guidelines,^13-15^ dado o grande número de combinações possíveis de lesões valvares e a dificuldade de padronização das condutas. Observamos que, ao se tratar uma estenose aórtica importante, reduz-se a pressão de enchimento ventricular e, como consequência, após alguns dias, pode haver reversão ou melhora da estenose mitral diante da nova configuração hemodinâmica com o implante da prótese aórtica transcateter. Assim, é indispensável uma nova avaliação da “valva não culpada” para entendermos se a intervenção ainda é necessária ou se a lesão poderia ser a responsável pela manutenção de parte da sintomatologia.

Na esteira desse conceito, apresentamos alguns cenários hipotéticos em que o tratamento estagiado ou hierárquico da “valva culpada” poderia ser útil aos pacientes com risco aumentado para a cirurgia convencional (Tabela 1).

**Tabela 1:** – Cenários hipotéticos para o tratamento estagiado na doença valvar múltipla


Estenose aórtica importante e bom candidato à TAVI com: Insuficiência coronariana obstrutiva estável Insuficiência mitral moderada a importante Estenose mitral moderada a importante Insuficiência tricúspide moderada a importante
Insuficiência mitral importante e bom candidato ao reparo borda a borda por cateter com: Insuficiência tricúspide importante
Estenose mitral reumática importante e bom candidato à valvuloplastia mitral por cateter balão com: Insuficiência tricúspide importante

TAVI: implante percutâneo da válvula aórtica, do inglês transcatheter aortic valve implantion.

Em casos selecionados, o tratamento combinado transcateter mostra-se como uma alternativa razoável e apresenta resultados promissores no curto e no médio prazos.^16^ A melhor abordagem e a ordem de tratamento são objeto de estudo devido ao número limitado de casos na literatura e devem, portanto, ser avaliadas caso a caso.

Concluímos que a decisão terapêutica para pacientes com doença valvar, particularmente estenose aórtica, e com alto risco cirúrgico, deve levar em conta a identificação da “doença principal”, ou seja, aquela que está efetivamente causando os sintomas e/ou que pode impactar negativamente no prognóstico precoce. Tratamentos agressivos em pacientes idosos, frágeis e/ou com múltiplas comorbidades podem resultar em desfechos catastróficos. O conceito de valva culpada visa racionalizar a decisão clínica baseada na redução do risco, optando pelo tratamento menos invasivo, com máxima eficácia no alívio dos sintomas, na redução da morbimortalidade e na melhora da qualidade de vida dos pacientes.
