# Supplementary material for: Valva Culpada: Um Novo Conceito para Redução do Risco da Intervenção no Portador de Doença Valvar Grave
Source: Arq Bras Cardiol. 2025 Apr 17;122(4):e20240492. [Article in Portuguese] doi: 10.36660/abc.20240492 (PMC12217924; doi:10.36660/abc.20240492)
Supplement: Translation

# Translation: The Culprit Valve: A New Concept for Reducing the Risk of Intervention in Patients with Severe Valve Disease

**DOI:** 10.36660/abc.20240492i

**Authors:** Roberto Cintra de Azevedo Aragão, José Augusto Soares Barreto, Roney Orismar Sampaio

**Affiliations:** 1 Brazil Universidade Federal de Sergipe, São Cristóvão, SE – Brazil; 2 Brazil Fundação São Lucas, Centro de Ensino e Pesquisa, Aracaju, SE – Brazil; 3 Brazil Universidade Federal de Sergipe - Programa de Pós-Graduação em Ciências da Saúde, Aracaju, SE – Brazil; 4 Brazil Instituto do Coração do Hospital das Clínicas da Faculdade de Medicina da Universidade de São Paulo, São Paulo, SP – Brazil

**Keywords:** Heart Valve Diseases, Prognosis, Cardiology

In clinical practice, it is not unusual to come across two relatively serious clinical conditions that occur concomitantly and may justify the patient’s symptoms. For example, associations between aortic stenosis and coronary artery disease, or between aortic stenosis and primary or secondary mitral regurgitation, both moderate to severe, are common in the real world. In the case of patients with low surgical risk, combined treatment is a consensus at the time of surgical intervention despite the consequences of these combined procedures, such as increased cardiovascular morbidity and mortality. For example, aortic valve (AV) replacement, when combined with coronary artery bypass graft surgery, increases the risk of mortality by almost twofold, just as AV replacement, when combined with mitral valve (MiV) surgery, increases this risk by more than threefold.^1^ However, in patients with high perioperative risk, especially when a less invasive isolated procedure can be used, the decision to indicate a broader treatment but with higher morbidity and mortality versus an isolated treatment but with lower morbidity and mortality is not trivial.

Recently, in Heart Team (HT) discussions, thanks to the advent of AV treatment by transcatheter aortic valve implantion (TAVI) or even MiV “edge-to-edge” repair and “valve in valve” procedure, the idea that the isolated treatment of the most severe lesion, which is likely the main cause of the patient’s symptoms, can optimize the patient’s outcome, reducing the expected morbidity and mortality and substantially improving the patient’s quality of life, has gained momentum.

In acute coronary syndrome (ACS), it is common practice to treat the artery responsible for the acute event first. At a second stage, which may occur during hospital admission or after stabilization of the clinical condition, additional revascularization of other obstructive coronary lesions may or may not be indicated. Extrapolating to valve diseases, the concept of “culprit valve” and its hierarchically prioritized treatment have emerged. In Heart Team discussions, it is not uncommon to face situations of this type; therefore, establishing the concept of the “culprit valve,” as is the case with ACS, can bring tangible benefits to these patients.

Multiple valve disease is a highly prevalent condition, and its incidence has increased with population aging. The disease is acquired most of the time and has been associated with a worse prognosis.^2^ Furthermore, in countries where rheumatic valve disease is prevalent, the incidence of simultaneous severe multivalvular disease is even higher.^3^

The methods used to quantify valvular stenosis and regurgitation were validated for isolated valvular diseases, making the analysis of multiple diseases difficult and, therefore, posing a diagnostic and therapeutic challenge.^4^ Hemodynamic interactions depend on specific combinations of valve lesions, severity, time of onset of each individual lesion, overload conditions, and ventricular systolic performance. The main conditions that may impact the diagnosis of multiple valve disease are: the frequent presence of low-flow and low-gradient stenosis; the inapplicability of the continuity equation when transvalvular flows are unequal; and severe valve lesions, which may induce or increase secondary mitral and tricuspid regurgitation upstream. Besides, methods derived from pressure half-time may be invalid in the presence of altered left ventricular compliance/relaxation.^2^

As an example, functional mitral regurgitation is present in more than 60% of the patients with aortic stenosis. It reduces afterload and systolic volume, which may favor a condition of lower flow and low gradient with the risk of underestimating the severity of aortic stenosis. At the same time, the presence of mitral regurgitation reduces total afterload and increases ejection fraction, which may hide subclinical myocardial dysfunction. In these cases, the AV area appears to be the most reliable method of assessment.^4^

In such a scenario, multiple valve disease can be difficult to interpret, requiring multimodal imaging and a Heart Team for a better diagnostic strategy and therapeutic management, especially with the progressive evolution of catheter procedures.^2,4^

Combined valve surgery displays a high hospital mortality rate, especially in patients with left ventricular dysfunction, renal failure, and older age, a population in which the presence of multiple valve disease is not uncommon.^5-7^ Patients’ preferences should also be a variable when choosing the path to follow. A more holistic decision-making that considers all of these factors can bring better results in the short, medium, and long term.

In the post-TAVI hemodynamic assessment of patients with severe aortic stenosis and concomitant mitral stenosis (mitral valve area (MVA) ≤ 2.0 cm^2^), half of the patients improved the MVA beyond 2.0 cm,^2^ which may allow one to conclude that it had been overestimated (“pseudo” mitral stenosis). Some predictors suggesting the presence of true mitral stenosis are: MVA ≤ 1.5 cm,^2^ mitral annular distension ≤ 1 mm, or extension of calcification to the anterior and posterior mitral leaflets.^8^ Therefore, better identification of the patients who may benefit from combined treatment could reduce morbidity and mortality and avoid unnecessary multiple treatments.

On the other hand, not correcting an associated moderate/major valve lesion, a situation that is increasingly common in elderly patients with aortic stenosis who undergo TAVI, requires an integration of factors and individualization. A comprehension of the natural history, etiology of the affected valves, life expectancy, comorbidities, surgical risk, the possibility of repair, and feasibility of the percutaneous approach are critical for decision-making. Hemodynamic interactions can alter the clinical expression of each unique lesion; hence, clinicians should be aware of the interactions that may impact the diagnosis. All of these factors must be considered in the valve analysis by the Heart Team.

Elderia et al. showed that a staged interventional approach has a short-term survival advantage compared to combined surgery for the treatment of coronary artery disease and aortic stenosis.^9^ A recent study has shown that percutaneous coronary intervention after TAVI does not expose patients to greater periprocedural risks and provides a trend of favorable clinical results in the medium and long term,^10^ even in the case of incomplete myocardial revascularization.^11^ Yet, despite the high success rate of post-TAVI coronary access, percutaneous coronary intervention (PCI) before TAVI should be considered in patients with severe proximal coronary artery disease (CAD), particularly acute coronary syndrome, angina symptoms or subocclusive lesions, especially if the selected prosthesis is supra-annular. Predicting the viability of future coronary access based on a pre-TAVI tomographic imaging assessment is key to selecting the prosthesis type and implant height and performing the commissural alignment, especially in young and lower-risk patients.^12^

The limited evidence on medical, surgical, and interventional treatment in the setting of multiple valve disease is emphasized by the existence of C-level evidence in most guidelines,^13-15^ given the large number of possible combinations of valve lesions and the difficulty in standardizing procedures. We observed that, when treating major aortic stenosis, the ventricular filling pressure is reduced and, as a consequence, after a few days, there may be reversal or improvement of mitral stenosis given the new hemodynamic configuration with the implantation of the transcatheter aortic prosthesis. Thus, a new evaluation of the “non-culprit valve” would be necessary to understand whether the intervention is still necessary or whether the lesion could be responsible for maintaining part of the symptoms.

Within this concept, we outline some hypothetical scenarios in which the staged or hierarchical treatment of the “culprit valve” could be useful for patients at increased risk for conventional surgery (Table 1).

**Table 1:** – Hypothetical scenarios for staged treatment in multiple valve disease


Severe aortic stenosis and a good candidate for TAVI with: Stable obstructive coronary insufficiency Moderate to severe mitral regurgitation Moderate to severe mitral stenosis Moderate to severe tricuspid regurgitation
Severe mitral regurgitation and a good candidate for edge-to-edge repair by catheter with: Severe tricuspid insufficiency
Significant rheumatic mitral stenosis and a good candidate for mitral valvuloplasty by balloon catheter with: Severe tricuspid insufficiency

TAVI: transcatheter aortic valve implantion.

In selected cases, combined transcatheter treatment has proven to be a reasonable alternative, with promising results in the short and medium term.^16^ The best approach and order of treatment are being studied due to the limited number of cases in the literature and, therefore, should be evaluated on a case-by-case basis.

In conclusion, the therapeutic decision-making for patients with valve disease, particularly aortic stenosis, as well as high-risk surgical patients, should take into account the identification of the “main disease,” i.e., the one that is actually causing the symptoms and/or that can negatively impact on early prognosis. Aggressive treatments in the elderly, frail patients, and/or patients with multiple comorbidities can result in catastrophic outcomes. The concept of culprit valve aims to rationalize clinical decision-making based on risk reduction, opting for the least invasive treatment, with maximum effectiveness in symptom relief, reduced morbidity and mortality, and better quality of life for patients.
